# Insights from Metabolomic and Transcriptomic Analyses into Sulforaphane’s Protective Mechanism Against Deoxynivalenol Toxicity via Spermine Regulation

**DOI:** 10.3390/toxins17040178

**Published:** 2025-04-03

**Authors:** Yeyi Xiao, Jianliang Wu, Menke Feng, Jie Wang, Lele Qi, Chao Xu, Haifei Wang, Wenbin Bao

**Affiliations:** 1 Key Laboratory for Animal Genetics, Breeding, Reproduction and Molecular Design, College of Animal Science and Technology, Yangzhou University, Yangzhou 225009, China; yeyixiao@126.com (Y.X.); 15736705690@163.com (M.F.); wangjie4228@163.com (J.W.); llqi1016@163.com (L.Q.); xc13952750145@163.com (C.X.); hyfiwang@yzu.edu.cn (H.W.); 2 Beijing Zhongyu Pig Breeding Co., Ltd., Beijing 100194, China; jl1617@163.com; 3 Joint International Research Laboratory of Agriculture and Agri-Product Safety, Yangzhou University, The Ministry of Education of China, Yangzhou 225000, China

**Keywords:** deoxynivalenol, sulforaphane, spermine, metabolite, oxidative stress

## Abstract

Deoxynivalenol (DON) is a mycotoxin ubiquitously present in the environment. Emerging evidence demonstrated that sulforaphane (SFN) exerts potent protective effects against DON-triggered cytotoxicity through multimodal mechanisms. This study aimed to investigate the protective mechanism of SFN during DON exposure. Untargeted metabolomics of IPEC-J2 cells revealed a total of 399 differential metabolites between the DON and control group and 365 differential metabolites between the SFN + DON and DON group. KEGG enrichment was performed to investigate the potential regulatory pathways. The transcriptome identified a total of 1839 differential expression genes (DEGs) between DON and SFN + DON groups. This result indicated that DON exposure and SFN treatment have a profound impact on cellular metabolism and genes. Integrated analysis of the transcriptome and metabolome showed that spermine was a potential biomarker for SFN treatment. SFN increased spermine abundance by regulating genes in glutathione, beta-alanine, and arginine and proline metabolism pathways. Functional experiments demonstrated that spermine alleviated DON-induced oxidative stress, as evidenced by increased cell viability, reduced ROS levels, restored mitochondrial membrane potential (ΔΨm), and normalized antioxidant enzyme activity. Moreover, spermine significantly decreased the cell apoptosis rate induced by DON, which suggested that spermine significantly alleviated the DON-induced cytotoxicity. Overall, these findings elucidated the protective role of SFN through spermine-related mechanisms against the toxicity of DON.

## 1. Introduction

Deoxynivalenol (DON) is a common mycotoxin found in grains. With the frequent occurrence of precipitation in southern China, the degree of DON contamination shows an increasing trend. As a secondary metabolite produced by Fusarium, there is an urgent need for control of DON pollution [1]. Mycotoxins present in animal feed have the potential to infiltrate the human body, posing a threat to public health [2]. The degradation of DON primarily involves physical, chemical, and biological methods. Physical detoxification methods include high-temperature inactivation. However, DON is highly thermally stable, and high temperatures can also affect the nutritional value of feed. Therefore, high-temperature detoxification is not the most ideal method for mycotoxin removal. Chemical methods refer to the use of chemical reagents to treat mycotoxins. However, this approach may result in residues in feed, posing potential risks to animal health. Among biological methods, the use of specific microorganisms or their enzymes to degrade mycotoxins represents a promising detoxification strategy. However, different microorganisms exhibit varying abilities to degrade different mycotoxins, and it is challenging to ensure that microorganisms can efficiently degrade mycotoxins in complex production environments [3]. DON causes various degrees of damage to animals. Pigs are more sensitive to DON compared to rodents and poultry [4]. In pigs, symptoms include reduced feed intake, weight loss, and decreased reproductive performance. When the diet contains 1–3 mg/kg of DON, the growth of pigs begins to decline. Particularly for naturally contaminated feed, 1–2 mg/kg of DON significantly reduces feed intake and weight gain of pigs [5]. The high susceptibility of pigs to DON is determined by their unique physiological structure and metabolic characteristics, which can be summarized as efficient absorption and inefficient metabolism. Upon ingestion by pigs, approximately 80% of DON is rapidly absorbed by the small intestine and enters the blood circulation system. Only a portion of DON reaching the liver and colon is converted into non-toxic metabolites, while the majority is excreted in the original form via urine and bile. This results in prolonged retention of the toxin in vivo, thereby further exacerbating the toxic effects of DON [4]. The high sensitivity of pigs to DON makes intestinal porcine epithelial cells (IPEC-J2) an ideal material for studying the mechanisms of DON [6,7]. IPEC-J2 cells as adherent monolayers cannot fully replicate the in vivo epithelial barrier function. However, this cell line is an internationally recognized model for studying DON-induced intestinal toxicity [8]. Its advantages lie in standardized procedures, high reproducibility, and ease of conducting mechanistic studies at the molecular level. The toxicity of DON is primarily attributed to its inhibitory effect on ribosomal protein biosynthesis. In murine macrophages, upon exposure to 250 ng/mL DON, a general decrease in the expression levels of translation-related proteins interacting with ribosomes was observed, indicating that the toxicity of DON disrupts normal protein synthesis processes. Recent research showed that DON induced severe DNA damage in IPEC-J2 cells, knockdown of DNA repair enzyme LIG3 exacerbated cell death and inflammation, while overexpression mitigated these effects [9]. In hepatocytes, 3-indole-propionic acid (IPA) reduced m6A modification and stability of Malat1 lncRNA by activating the m6A demethylase FTO, enhanced Nrf2-mediated antioxidant pathways, and thereby mitigated DON-induced hepatocellular oxidative stress and liver injury [10]. In recent years, studies have shown that mitochondria are the key molecular targets of DON [11]. The functional state of mitochondria, which serve as the energy factories of cells, is intricately linked to the intracellular levels of reactive oxygen species (ROS). DON activates NADPH oxidase, which subsequently catalyzes the electron transfer from NADPH to molecular oxygen, generating ROS, such as superoxide anions (O_2_^−^). Excessive accumulation of ROS compromises the integrity of the mitochondrial membrane, leading to increased membrane permeability and subsequent impairment of mitochondrial function [12].

To mitigate the damage induced by DON toxicity, plant extracts are regarded as potential additives for alleviating DON toxicity, owing to their antioxidant properties and safety. A recent study showed that lycopene alleviated the oxidative stress and reduced cell apoptosis induced by DON via regulating oxidative phosphorylation (OXPHOS) in IPEC-J2 cells [13]. Resveratrol was also reported to alleviate liver injury by activating the Nrf2/Keap1 signaling pathway induced by T-2 toxin in mice [14]. Curcumin, a non-toxic dietary compound, was considered a potential phytogenic feed additive due to its excellent antioxidant properties to mitigate mycotoxins [15]. Sulforaphane (SFN) is a natural compound with remarkable antioxidant properties. For instance, all four compounds activate antioxidant pathways, lycopene improves mitochondrial function, resveratrol up-regulates Nrf2/Keap1 signaling, curcumin scavenges ROS directly, and SFN also activates Nrf2/Keap1 signaling. This convergence of antioxidant activity highlights a conserved strategy among plant extracts to combat DON-induced oxidative stress in IPEC-J2 cells. SFN enhanced the antioxidant defense system by activating the Nrf2/HO1 pathway and attenuating inflammatory signaling [16]. SFN has also been shown to reduce ROS production and increase the activities of antioxidant enzymes [17]. Low-dose sulforaphane pretreatment protects cells from H_2_O)-induced damage. During this process, sulforaphane activates Nrf2 and increases intracellular glutathione (GSH) levels within 24 h [18]. It was demonstrated that, in a murine model of acute lung injury (ALI), SFN attenuated the severity of lipopolysaccharide (LPS)-induced ALI by inducing Nrf2 protein expression, confirming its effective protective role against ALI [19]. These findings indicated that the antioxidant activity of SFN may alleviate the oxidative stress induced by DON. Meanwhile, our previous data suggested that SFN alleviated the oxidative stress induced by DON both in vivo and in vitro by activating cellular autophagy, however, the underlying regulatory mechanisms still need to be further investigated [20].

In this study, integrated metabolomic and transcriptomic analyses identified spermine as the most discriminant metabolite in SFN-treated cells, suggesting a protective role of spermine against DON-induced cytotoxicity. As a ubiquitous endogenous polyamine, spermine serves as a pivotal regulator in counteracting oxidative stress. Its multiple amine groups directly neutralize free radicals through hydrogen atom donation, effectively quenching ROS by converting them into chemically inert compounds. This process mitigates the oxidative damage inflicted by free radicals on cellular components, including lipids, proteins, and DNA [21]. A previous study showed that spermine treatment significantly reduced the content of malondialdehyde (MDA) in the liver and spleen of mice, enhanced the activity of catalase (CAT) in the spleen, and increased the anti-hydroxyl radical (AHR) capacity, thereby eliminating free radicals and mitigating oxidative stress. Specifically, spermine enhanced the nuclear translocation of Nrf2, which increased the expression of heme oxygenase-1 (HO-1) and NADPH quinone oxidoreductase-1, and then decreased the production of ROS [22].

In this study, transcriptomics and metabolomics were employed to analyze the potential molecular mechanisms by which SFN alleviates DON oxidative stress and investigate the protective effect of spermine on DON toxicity. The study was conducted to explore the specific biological pathways and molecular mechanisms through which SFN mitigates the toxicity of DON. Our data offered a new perspective on DON’s cytotoxicity and its prevention strategies.

## 2. Results

### 2.1. Alterations in Cellular Metabolites Induced by SFN Treatment and DON Exposure

**Figure 1:**
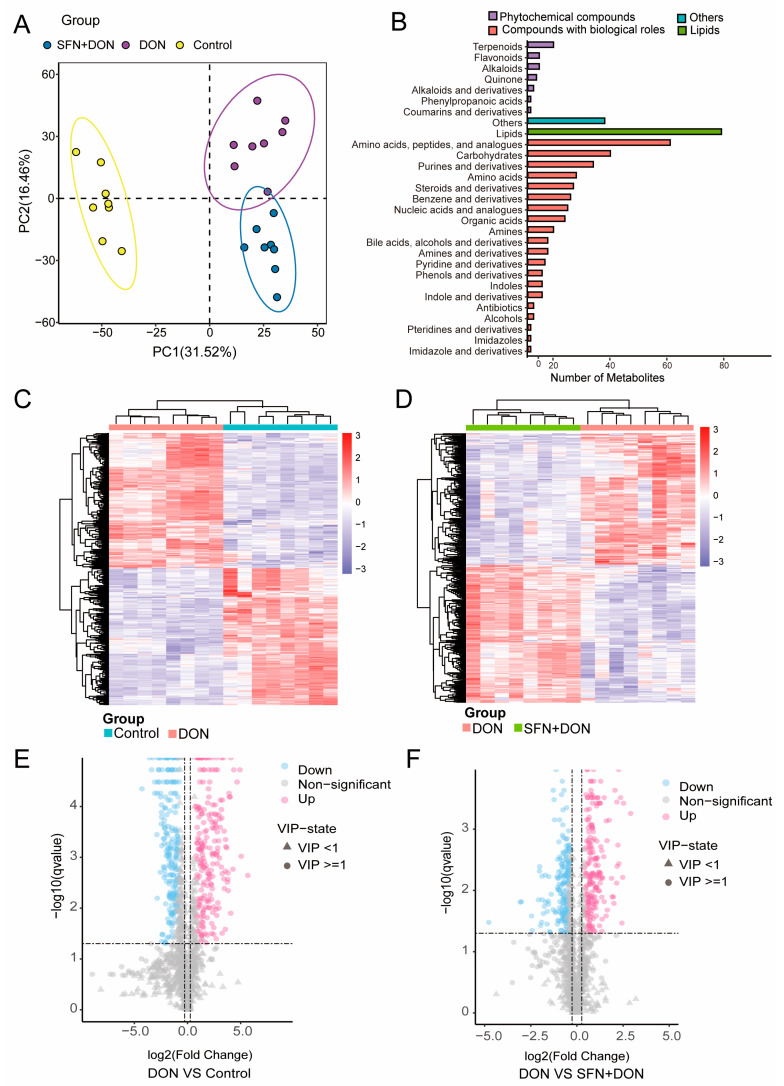
Metabolic analyses of cells upon SFN treatment and DON exposure. (**A**) PLS-DA for differential metabolites between different groups. (**B**) Classification of the identified metabolites. (**C**) Heatmap of cluster analysis of differential metabolites between DON and control group. (**D**) Heatmap of cluster analysis of differential metabolites between DON and SFN + DON group. The solid lines on the upper and left sides represent metabolites with the same expression pattern. (**E**) Volcano plot of differential metabolites between DON and control group. The dashed line represents the significance threshold. (**F**) Volcano plot of differential metabolites between DON and SFN + DON group. Control: cells without DON exposure; DON: cells exposed to 1 μg/mL DON; SFN + DON: cells treated with 2 μM SFN and 1 μg/mL DON.

Firstly, we performed untargeted metabolomics to further investigate the protective mechanism of SFN during DON exposure. Partial least squares discriminant analysis (PLS-DA) revealed clear metabolomic alterations between different groups (Figure 1A). Annotation for metabolites suggested that metabolites mainly belonged to phytochemical compounds, compounds with biological roles, and lipids, such as amino acids, peptides, analogues, and carbohydrates (Figure 1B). Hierarchical clustering was then performed to analyze the expression patterns of the differential metabolites (Figure 1C,D). A total of 399 differential metabolites (201 down-regulated and 198 up-regulated) were identified between the DON group and control groups (Figure 1E; Table S1). And a total of 365 differential metabolites (178 down-regulated and 187 up-regulated) were identified between the DON group and SFN + DON groups (Figure 1F; Table S2).

### 2.2. Analysis of Differential Metabolites Caused by SFN Treatment and DON Exposure

**Figure 2:**
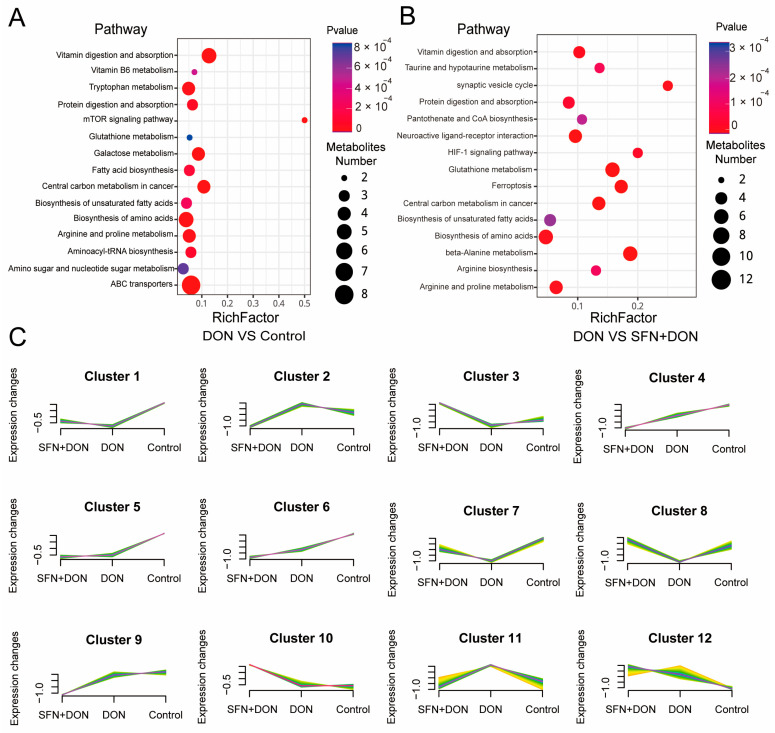
Analysis of different metabolites among different groups. (**A**) KEGG analysis of metabolites between DON and control group. (**B**) KEGG analysis of metabolites between DON and SFN + DON group. (**C**) Trend analysis of metabolites. Control: cells without DON exposure; DON: cells exposed to 1 μg/mL DON; SFN + DON: cells treated with 2 μM SFN and 1 μg/mL DON.

In order to further explore the regulatory mechanism of SFN, KEGG enrichment analysis was carried out for different metabolites. The results showed that the differential metabolites between the DON group and control group were enriched in pathways such as ABC transporters, biosynthesis of amino acids, and vitamin digestion and absorption (Figure 2A). Meanwhile, the differential metabolites between the DON group and SFN + DON group were mainly enriched in arginine and proline metabolism, biosynthesis of amino acids, and glutathione metabolism (Figure 2B). Then we analyzed the trends of metabolites among the three groups. The analysis revealed a total of 12 distinct trends. Three of these trends exhibited up-regulation following DON exposure and down-regulation subsequent to SFN treatment (Clusters 2, 9, and 11). Conversely, six trends demonstrated down-regulation after DON exposure and up-regulation upon SFN treatment (Clusters 1, 3, 5, 7, 8, and 10). Notably, three SFN treatments displayed trends identical to those observed during DON exposure (Clusters 4, 5, and 12; Figure 2C).

### 2.3. Integrated Analysis of Transcriptome and Metabolome

**Figure 3:**
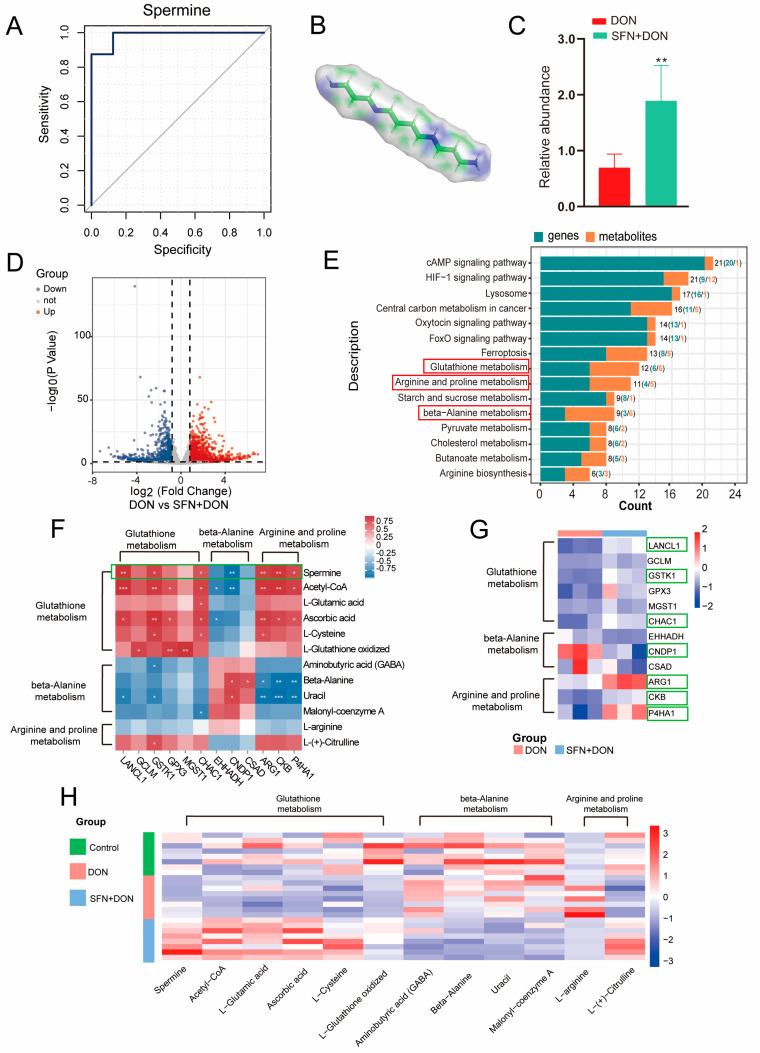
Comprehensive analysis of transcriptome and metabolome between DON and SFN + DON group. (**A**) ROC analysis. (**B**) The 3D structure of spermine. (**C**) Relative abundance of spermine in IPEC-J2 cells. DON: cells exposed to 1 μg/mL DON; SFN + DON: cells treated with 2 μM SFN and 1 μg/mL DON. (**D**) Volcano plot of DEGs between DON and SFN + DON group. The dashed line represents the significance threshold. (**E**) Comprehensive analysis of DEGs and differential metabolites between DON and SFN + DON group. The red box: pathways involved in spermine metabolism. (**F**) Correlations of DEGs and differential metabolites. * *p* < 0.05; ** *p* < 0.01; *** *p* < 0.001. (**G**) Heatmap of DEGs. The green box: genes significantly associated with spermine metabolism. (**H**) Heatmap of differential metabolites. Control: cells without DON exposure; DON: cells exposed to 1 μg/mL DON; SFN + DON: cells treated with 2 μM SFN and 1 μg/mL DON.

To further explore the protective mechanisms of SFN, we focused on the differential metabolites between the DON group and DON+SFN group. Using ROC analysis, we identified spermine as a potential biomarker for SFN treatment (Figure 3A). The 3D structure of spermine is shown in Figure 3B. Furthermore, we discovered that SFN evidently increased the abundance of spermine in IPEC-J2 cells (Figure 3C). To further investigate the role of spermine, we performed transcriptome sequencing analysis between DON and SFN + DON groups. The results showed a total of 1839 DEGs (738 down-regulated and 1101 up-regulated) were identified (Figure 3D; Table S3). Then, the results of comprehensive analysis suggested that glutathione metabolism, beta-alanine metabolism, and arginine and proline metabolism were enriched, all of which involve spermine (Figure 3E). To better understand the mechanism, we analyzed the correlation between differential genes and metabolites involved in glutathione metabolism, beta-alanine metabolism, and arginine and proline metabolism pathways. The results showed that the abundance of spermine was positively correlated with the expression of multiple genes (LANCL1, GSTK1, CHAC1, ARG1, CKB, and P4HA1) and negatively correlated with CNDP1 (Figure 3F). In addition, transcriptomics data demonstrated that SFN supplementation increased the expression levels of genes positively correlated with the abundance of spermine and decreased the expression level of CNDP1 which is negatively correlated with spermine abundance (Figure 3G). The heatmap of metabolite abundance also showed a consistent trend with the gene heatmap (Figure 3H). These results suggested that SFN increased spermine abundance by regulating the expression of genes involved in the glutathione metabolism, beta-alanine metabolism, and arginine and proline metabolism pathways.

### 2.4. Spermine Alleviates Oxidative Stress Induced by DON

**Figure 4:**
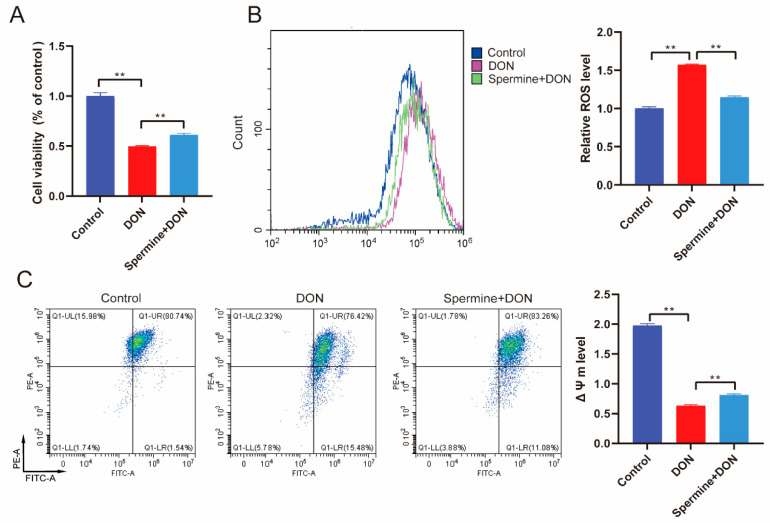
The role of spermine in DON-induced oxidative stress. (**A**) Cell viability detection upon spermine treatment and DON exposure. (**B**) ROS levels after spermine treatment during DON exposure. (**C**) Detection of ΔΨm level after spermine treatment and DON exposure. Control: cells without DON exposure; DON: cells exposed to 1 μg/mL DON; Spermine+DON: cells treated with 1 μM spermine and 1 μg/mL DON. Data are shown as mean ± SD, ** *p* < 0.01. n = 3 per group.

**Figure 5:**
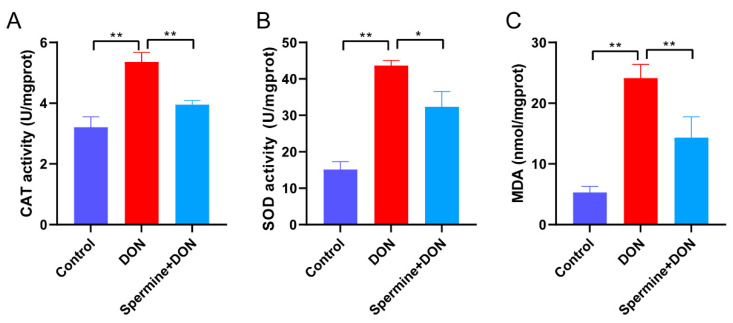
Effect of spermine on antioxidant enzyme activity. (**A**) Detection of CAT activity upon spermine treatment and DON exposure. (**B**) Detection of SOD activity after spermine treatment during DON exposure. (**C**) Detection of MDA level after spermine treatment and DON exposure. Control: cells without DON exposure; DON: cells exposed to 1 μg/mL DON; Spermine + DON: cells treated with 1 μM spermine and 1 μg/mL DON. Data are shown as mean ± SD, * *p* < 0.05, ** *p* < 0.01. n = 3 per group.

Given the research we have discussed above, we hypothesized that spermine could protect cells from DON toxicity. To test this, we treated DON-exposed cells with spermine, which resulted in significant increases in cell viability compared with the DON group (Figure 4A). We then assessed ROS levels and, as hypothesized, spermine treatment led to a reduction in ROS (Figure 4B). The detection of ΔΨm level also suggested that spermine restored the decrease in ΔΨm level induced by DON, indicating that spermine supplementation alleviates DON-induced oxidative stress (Figure 4C). To further verify the antioxidant activity of spermine, we detected the activity of antioxidant enzymes (CAT and SOD) and the content of MDA. We found that spermine effectively restored the DON-induced elevation in antioxidant enzyme activity and decreased MDA levels (Figure 5). These results suggested that spermine significantly alleviated oxidative stress upon DON exposure.

### 2.5. Spermine Alleviates Cell Apoptosis Induced by DON

**Figure 6:**
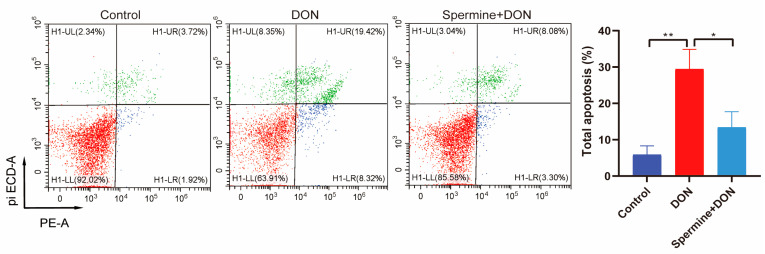
The role of spermine in DON-induced apoptosis. Control: cells without DON exposure; DON: cells exposed to 1 μg/mL DON; Spermine + DON: cells treated with 1 μM spermine and 1 μg/mL DON. Data are shown as mean ± SD, * *p* < 0.05, ** *p* < 0.01. n = 3 per group.

We also measured the apoptosis rate of cells with spermine treatment and DON exposure. The data showed that spermine significantly decreased the cell apoptosis (Figure 6).

## 3. Discussion

DON pollution exhibits numerous characteristics, yet existing detoxification methods fail to entirely eliminate its toxic effects. Therefore, in recent years, numerous reports have focused on using food additives to alleviate the damage caused by DON [13,23]. We found that SFN significantly alleviated DON-induced toxicity, yet the potential mechanisms and landmark target sites of the protective effect of SFN remain to be elucidated. Acute exposure to high concentrations of DON induces evident toxic symptoms in pigs, while long-term intake of low concentrations of DON impairs both growth and reproductive performance in swine populations. In this study, the DON dose (1 μg/mL) was determined based on preliminary experiment results [6,24]. The experiment showed that this concentration could induce significant inflammation and cell damage, making it suitable for mechanistic studies. Based on our previous findings demonstrating that SFN treatment did not significantly affect cellular viability or apoptotic rates, the current investigation specifically focused on the DON-exposed group and the SFN + DON cotreatment group. This experimental design was strategically adopted to elucidate the underlying protective mechanisms of SFN against DON-induced cytotoxicity. Herein, to further elaborate on this issue, we performed untargeted metabolomics analysis. The results revealed significant metabolomic alterations among different groups. The large number of differential metabolites identified between the DON and control group, as well as between the DON and SFN + DON group, indicated that DON exposure and SFN treatment have a profound impact on cellular metabolism. The KEGG enrichment analysis further demonstrated that these differential metabolites were involved in various important metabolic pathways. Among them, we noted that, after SFN treatment, the differential metabolites were enriched in pathways such as ferroptosis and beta-alanine metabolism, which were not enriched between DON and control groups. Ferroptosis is an iron-dependent form of regulated cell death characterized by lipid peroxidation and GSH depletion. It is tightly linked to oxidative stress, as excessive ROS generation leads to accumulation of peroxidized lipids, particularly polyunsaturated fatty acids (PUFAs), which trigger ferroptotic cell death. Ferroptosis has been reported to participate in DON-induced oxidative stress by regulating the concentration of Fe^2+^ [25]. Our data indicated that ferroptosis could be a potential mechanism underlying the antioxidant effect of SFN. β-Alanine, also known as 3-aminopropanoic acid, is a non-protein amino acid that plays a crucial role in various biological processes [26]. Following its condensation with β-alanine to form pantothenic acid, pantothenate is initially converted to 4′-phosphopantothenate via pantothenate kinase-catalyzed phosphorylation. This intermediate subsequently combines with cysteine to generate 4′-phosphopantothenoylcysteine, which undergoes decarboxylation and ATP-dependent phosphorylation to yield a coenzyme (CoA) [27]. It is a key component in the synthesis of pantothenate, which is essential for the production of CoA. CoA is involved in over 100 metabolic reactions in the body, highlighting the importance of β-alanine in cellular metabolism [28]. β-Alanine has been shown to enhance the antioxidant capacity of cells by increasing the production of glutathione, a key antioxidant. Meanwhile, β-alanine also reduces the levels of ROS and protect cells from oxidative stress [29]. These findings suggested that SFN modulates multiple metabolic pathways to mitigate the toxic effects of DON.

Using a comprehensive approach, we first identified spermine, a polyamine known for its role in cellular homeostasis and stress response, as a potential biomarker for SFN treatment. Our findings further demonstrated that SFN supplementation significantly increased spermine abundance during DON exposure. Spermine is a natural product of cellular metabolism which regulates cell proliferation, differentiation, and apoptosis [30]. It has been reported that spermine could scavenge ROS and alleviate oxidative stress in mouse myocardial cells [31]. In this study, we found that spermine treatment enhanced cell viability and reduced ROS accumulation and apoptosis rates in DON-exposed cells. The antioxidant capacity of spermine stems from its improvement of mitochondrial function, evidenced by the recovered ΔΨm level. This result is consistent with the existing research results [32,33].

To gain deeper insight into the mechanism, we further analyzed the correlation between differential gene expression and metabolic pathways linked to spermine biosynthesis. Our data revealed SFN enhances spermine levels by regulating several metabolic pathways, including glutathione metabolism, beta-alanine metabolism, and arginine and proline metabolism. Glutathione (GSH) plays a significant role in maintaining redox balance and protecting cells from oxidative stress. However, the antioxidative function of glutathione metabolism might act indirectly rather than directly eliminating ROS. Studies have shown that glutathione seems to play a role in protecting cells from the cytotoxicity of spermine-derived aldehydes [34], which indicated that the glutathione metabolic pathway holds the potential to diminish toxic metabolites generated by spermine during ROS clearance. Spermine influences beta-alanine metabolism through its role in polyamine metabolism. Specifically, spermine can be broken down by polyamine oxidases to produce 3-aminopropanal, which is a precursor to beta-alanine [35]. β-Alanine serves as a precursor for pantothenate, which is essential for CoA biosynthesis. CoA is required for activating S-adenosylmethionine (SAM), a key methyl donor in polyamine synthesis [36]. Meanwhile, β-alanine metabolism may affect the availability of substrates for polyamine synthesis by regulating intracellular amino acid pools (such as histidine) and indirectly regulate the level of spermine. Arginine metabolism provides a precursor for polyamine synthesis. Specifically, arginine is converted to ornithine by arginase (ARG1), which is hydrolyzed by ornithine decarboxylase (ODC) to produce putrescine, and finally arginine is synthesized [37]. Herein, we found that ARG1 was significantly up-regulated which indicated that arginine and proline metabolism is the primary route through which SFN mediates the up-regulation of spermine. These three metabolic pathways form a tight functional network through substrate sharing, antioxidant synergy, and gene expression regulation. As a core metabolite, spermine not only directly eliminates ROS but also enhances the overall defense ability of cells against oxidative stress by regulating key genes in other metabolic pathways. This mechanism plays a crucial role in the mechanism of SFN alleviating DON toxicity, providing a theoretical basis for detoxification strategies targeting polyamine metabolism.

## 4. Conclusions

In summary, this study provides comprehensive insights into the molecular mechanisms underlying the protective effects of SFN against DON-induced toxicity, emphasizing the crucial role of spermine in alleviating oxidative stress and cell apoptosis. This research not only advances our understanding of SFN’s therapeutic potential but also offers a theoretical foundation for developing polyamine-targeted interventions to combat mycotoxin-related health risks. Based on this study, polyamine supplements for animal feed or human nutrition could be developed to mitigate deoxynivalenol (DON) risks.

## 5. Materials and Methods

### 5.1. Chemicals

Deoxynivalenol (DON, purity: ≥99%, MSS1011) was purchased from Pribolab (Qingdao, China). Spermine (purity: ≥99.81%, HY-B1777) and sulforaphane (SFN, purity: 99.75%, HY-13755) were purchased from MedChemExpress (MCE, Shanghai, China).

### 5.2. Cell Treatment

IPEC-J2 cells were cultured in 6-well plates at 5.0 × 10^5^ cells per well with Dulbecco’s modified Eagle’s medium (DMEM:F12 medium = 1:1, Gibco, 11965084, New York, NY, USA) containing 10% fetal bovine serum (Gibco, A5670401, New York, NY, USA). The density of adherent cells reached 80%. After 24 h, cells from the control group were incubated with completed DMEM, cells from the DON group were exposed to 1 μg/mL DON for 24 h, and cells from Spermine + DON group were treated with 1 μM spermine with DON exposure for 24 h [38]. For SFN treatment, control: cells without DON exposure; DON: cells exposed to 1 μg/mL DON; SFN + DON: cells treated with 2 μM SFN and 1 μg/mL DON [14].

### 5.3. Transcriptome Analysis

Three biological replicates of SFN- and DON-treated cells were collected for transcriptome analysis. Total RNA of cells was isolated using Trizol reagent (Takara, 9108Q, Dalian, China). Then, the quality and quantity of RNA were assessed using a Qubit 4 Fluorometer (Thermo Fisher Scientific, Waltham, MA, USA). The quality and quantity control standards for RNA were rRNA ratio (28 s/18 s) > 2.6 and RNA integrity number (RIN) > 9.0. mRNA was then purified from total RNA and fragmented. Double-stranded cDNA was synthesized from the fragmented mRNA. The cDNA library was constructed by end-repair, A-tail addition, and adapter ligation, followed by PCR amplification. Sequencing was performed on an Illumina Hiseq-PE150 high-throughput sequencing platform. Genes with |log2-fold change| ≥ 0.8 and a corrected *p*-value < 0.05 were identified as DEGs.

### 5.4. Metabolomic Analysis

Eight biological replicates of SFN- and DON-treated cells were collected for metabolomics detection. Metabolite extraction and LC-MS/MS analysis were performed by BGI-Shenzhen (Shenzhen, China). Electrospray ionization (ESI) was employed with positive/negative ion mode switching to cover polar and non-polar metabolites. Mass range: *m*/*z* 100–1000. Chromatographic separation was achieved on a Hypesil Gold C18 column (2.1 × 100 mm, 1.9 μm) maintained at 40 °C, with a mobile phase consisting of (A): 0.1% aqueous formic acid and (B): 0.1% formic acid in methanol. The gradient elution protocol was programmed as follows: initial 2% B (1.5 min), linear increase to 100% B over 12 min, isocratic hold at 100% B for 0.5 min (14 min), followed by re-equilibration to initial conditions (14 min). Mass spectrometric detection employed alternating positive/negative ionization modes with optimized parameters: electrospray voltage 3.2 kV, capillary temperature 320 °C, sheath gas flow 40 arbitrary units (arb), and auxiliary gas flow 10 arb. Raw data processing, including peak alignment and metabolite quantification, was conducted using the Compound Discoverer 3.1 software. Qualitative metabolite analysis was conducted by integrating peak detection with the mzCloud, mzVault, and Masslist databases to identify and quantify the compounds. A comprehensive multivariate statistical analysis was carried out to explore metabolic variations and correlations across various groups. This analysis included partial least squares discriminant analysis (PLS-DA), hierarchical clustering, and metabolite correlation assessment. Differentially expressed metabolites were identified with VIP > 1, *p* < 0.05 between the two groups.

### 5.5. Comprehensive Analysis of Transcriptomics and Metabolomics

Integrated correlation networks between transcriptomic and metabolomic profiles were constructed through Pearson correlation analysis using MetaboAnalyst 5.0 https://www.metaboanalyst.ca/ (accessed on 2 April 2025). And *p* < 0.05 was considered to indicate a significant correlation. All of the differently expressed genes and metabolites were annotated according to the KEGG pathway database https://www.kegg.jp/kegg/pathway.html (accessed on 2 April 2025). Pathways containing differently expressed genes and metabolites were annotated according to the common pathways [39].

### 5.6. Analysis of Flow Cytometry

Cells were cultured in 6-well plates for 24 h and, after DON and spermine treatment, cells were collected for further analysis. Analysis of ROS level was determined using dihydroethidium (DHE, KeyGEN BioTECH, KGA7502-5, Nanjing, China). Cells were incubated with 10 μM of DHE at a temperature of 37 °C for 20 min. Subsequently, cells were washed three times with phosphate-buffered saline (PBS). Then cells were detected using a flow cytometer (Beckman Coulter, Brea, CA, USA). To assess the apoptosis rates, cells were treated with Annexin VPE/7-AAD (Solarbio, CA1030, Beijing, China). After staining, a flow cytometer (specifically, a Beckman Coulter device from Brea, CA, USA) was employed to conduct the analysis. The analysis of mitochondrial membrane potential (ΔΨm) was performed using a JC-1 dye detection kit (Beyotime, C2003S, Jiangsu, China). Cells were collected and then incubated with JC-1 at 37 °C for 20 min. After the incubation, cells were washed three times using PBS. Eventually, the ΔΨm was measured using FACS analysis (Beckman Coulter, Brea, CA, USA).

### 5.7. Analysis of Cell Viability

Cells were cultured in 96-well plates for 24 h before treatment. After treatment with 1 μg/mL DON and 1 μM spermine, CCK8 solution (YEASEN, 40203ES60, Shanghai, China) was dissolved in the medium and then added to each well. Subsequently, the plates were incubated at 37 °C. After a 2 h incubation, the absorbance at a wavelength of 450 nm was measured using a Tecan Infinite 200 microplate reader (Sunrise model, manufactured by Tecan in Switzerland).

### 5.8. Measurement of Antioxidant Parameters

Cells were cultured in 6-well plates for 24 h and then treated with DON and spermine as described above. After the treatment, cells were collected and washed using PBS before detection. The contents of SOD (cat. A001-2-2), CAT (cat. A007-1-1), and MDA (cat. A003-1-2) were analyzed via manufacturer-recommended procedures using specialized kits (Jiancheng Bioengineering, Nanjing, China).

### 5.9. Data Analysis

One-way ANOVA was implemented via SPSS 22.0 software. Levene’s test was used by default in SPSS analysis to assess homogeneity of variance. If heteroscedasticity was detected, Welch’s correction or non-parametric tests were applied prior to ANOVA. The Pearson correlation coefficient between differentially expressed genes (DEGs) and differential metabolites was analyzed through an online platform https://www.omicshare.com/ (accessed on 2 April 2025). The data were presented as means ± standard deviation (SD). Statistical significance was set at * *p* < 0.05 and ** *p* < 0.01.

## Data Availability

The original contributions presented in this study are included in the article and Supplementary Materials. Further inquiries can be directed to the corresponding author.

## Supplementary Materials

The following supporting information can be downloaded at: https://www.mdpi.com/article/10.3390/toxins17040178/s1, Table S1: Differential metabolites between DON and control group; Table S2: Differential metabolites between DON and SFN + DON group; Table S3: DEGs between DON and SFN + DON group.
